# Potential Role of Macrophage Phenotypes and CCL2 in the Pathogenesis of Takayasu Arteritis

**DOI:** 10.3389/fimmu.2021.646516

**Published:** 2021-05-17

**Authors:** Xiufang Kong, Ming Xu, Xiaomeng Cui, Lingying Ma, Huiyong Cheng, Jun Hou, Xiaoning Sun, Lili Ma, Lindi Jiang

**Affiliations:** ^1^ Department of Rheumatology, Zhongshan Hospital, Fudan University, Shanghai, China; ^2^ Department of Urology, Zhongshan Hospital, Fudan University, Shanghai, China; ^3^ Shanghai Key Laboratory of Organ Transplantation, Zhongshan Hospital, Fudan University, Shanghai, China; ^4^ Department of Cardiac Surgery, Shanghai Institute of Cardiovascular Diseases, Zhongshan Hospital, Fudan University, Shanghai, China; ^5^ Center of Clinical Epidemiology and Evidence-based Medicine, Fudan University, Shanghai, China

**Keywords:** Takayasu arteritis, macrophage phenotype, vascular fibrosis, biomarker, CCL2

## Abstract

**Objectives:**

To investigate vascular macrophage phenotype as well as vascular and peripheral chemokine (C-C motif) ligand 2 (CCL2) expression during different stages of disease progression in patients with Takayasu Arteritis (TA).

**Methods:**

In this study, 74 patients with TA and 50 controls were recruited. TA disease activity was evaluated with Kerr scores. Macrophage phenotype and CCL2 expression were examined by immunohistochemistry in vascular specimens from 8 untreated and 7 treated TA patients, along with 4 healthy controls. Serum CCL2 were quantified by enzyme-linked immune-absorbent assay from TA patients at baseline (n=59), at 6-months (n=38), and from 46 healthy volunteers. Vascular macrophage phenotype, vascular CCL2 expression and serum CCL2 levels during different stages, as well as the relationship between serum CCL2 and disease activity or other inflammatory parameters (erythrocyte sedimentation rate (ESR), C-reactive protein (CRP), and interleukin 6 (IL-6)) were investigated.

**Results:**

In untreated patients, vascular M1 macrophages and CCL2 showed increased expression, mainly in the adventitia. In contrast, in treated patients, vascular adventitial M1 and CCL2 expression were decreased, while vascular medial M2 macrophages and CCL2 levels were increased. Distribution of macrophages and CCL2 was consistent within the TA vascular lesions regardless of the disease stage. Furthermore, peripheral CCL2 was elevated in patients with TA (TA: 160.30 ± 120.05 vs. Control: 65.58 ± 54.56 pg/ml, P < 0.001). CCL2 levels were found to correlate with ESR, CRP, and IL-6 (all R values between 0.55 and 0.6, all P < 0.001). Receiver operating curve analysis demonstrated that CCL2 (at the cut-off value of 100.36 pg/ml) was able to predict disease activity (area under the curve = 0.74, P = 0.03). Decrease in CCL2 level was observed in patients with clinical remission (CR), but not in patients without CR, after 6 months of treatment (CR patients: baseline 220.18 ± 222.69 vs. post-treatment 88.71 ± 55.89 pg/ml, P = 0.04; non-CR patients: baseline 142.45 ± 104.76 vs. post-treatment 279.49 ± 229.46 pg/ml, P = 0.02).

**Conclusions:**

Macrophages contribute to vascular pathological changes in TA by undergoing phenotype transformation. CCL2 is an important factor for recruiting macrophages and a potential biomarker for disease activity.

## Introduction

Takayasu arteritis (TA) is a type of chronic granulomatous arteritis that involves the aorta and its main branches. In Asian population, it predominantly occurs in young women aged less than 40 years old (1, 2). Histologically, TA is characterized by vascular inflammation in the active stage and vascular fibrosis and remodeling in the chronic stage, which leads to irreversible vascular stenosis or even occlusion (3). Although current treatment regimens including glucocorticoids and immunosuppressants can achieve rapid relief of systemic inflammation, they are not believed to effectively halt tissue fibrosis, which may eventually lead to a poor prognosis. Therefore, clarification of the pathogenesis of TA is critical to develop effective treatment strategies.

acrophages are crucial immune cells, which are highly heterogenic and can be polarized into M1 or M2 phenotypes according to their functions. Both phenotypes play distinct roles in the pathogenesis of inflammatory disorders (4–8). Macrophage has been studied in vascular lesions of TA, and M2 phenotype was found to be dominated in the vascular lesions, but the impact of treatment on the macrophage phenotype was not fully illustrated (9). Chemokine (C-C motif) ligand 2 (CCL2) is a major monocyte chemotactic protein produced by macrophages as well as other cells such as endothelial cells, smooth muscle cells and fibroblasts (10). Production of this protein can be induced by pro-inflammatory cytokines such as interleukin (IL)-6 and tumor necrosis factor (TNF)-α, growth factors, or other antigen stimulants (11). CCL2 is elevated in the peripheral blood in patients with TA (12). However, its expression in the vascular lesions at the different stages of TA remains unclear.

This study aimed to compare the vascular macrophage phenotype as well as the vascular and peripheral expression of CCL2 between untreated and treated patients with TA.

## Materials and Methods

### Study Population

This study was performed in Zhongshan Hospital, Fudan University in China. Seventy-four patients with TA and 50 healthy controls were recruited from January 1, 2019 to May 31, 2020. Patients were diagnosed according to the 1990 American College of Rheumatology (ACR) classification criteria (13). Among them, 15 patients and 4 control subjects were enrolled for vascular tissue examination, while the remaining 59 patients with TA and 46 healthy control subjects were recruited for serum examination. Among the 15 patients who provided vascular tissue for histological examination, 8 were treatment-naive, and 7 had accepted medical treatment when they underwent surgery. Control samples of vascular tissue were obtained either from heart transplantation donors (normal aorta samples) or from patients undergoing nephrectomy (normal renal artery samples). Healthy controls for serum detection were included from the health examination center in our hospital. The study design and flowchart of this study were shown in **Supplementary Figure 1**.

The study and all its protocols were approved by the Institutional Review Board of Zhongshan Hospital, Fudan University, China (approval number: B2016-168), and conformed to the tenets of the Declaration of Helsinki. Written informed consents were obtained from all patients.

### Data and Specimen Collection

All clinical data (including symptoms, laboratory results, and imaging features) were collected at diagnosis and after 6 months of treatment for patients who provided serum samples. After treatment, clinical remission (CR) was considered if patients’ Kerr criteria was < 2. Otherwise, patients with Kerr ≥ 2 were assessed as non-CR. For patients who provided tissue samples, information about treatment regimen, clinical symptoms, laboratory reports, and imaging results were collected at the time of surgery.

### Immunohistochemistry

Specimens obtained from TA patients and four vascular controls were subjected to immunohistochemical (IHC) staining using a previously described method (14). In brief, tissue sections were deparaffinized and rehydrated. Antigen retrieval was conducted using citrate buffer solution (0.01 mol/L, pH 6.0). Endogenous peroxidase activity was blocked with 3% H_2_O_2_ (30 min, room temperature). Slides were blocked with 75 µL goat serum (30 min, RT). Subsequently, primary antibodies against CD68 (Abcam, ab955), HLA-DR (Abcam, ab92511), CD163 (Abcam ab182422) and CCL2 (Proteintech, 25542-1-AP) were added to each slide. The following day, the corresponding secondary antibodies were added to the slides and developed with 3,3ʹ-diaminobenzidine (DAB). The tissue sections of each patient were also subjected to routine hematoxylin and eosin (H&E) staining.

For the H&E or IHC analysis, all the slides were digitally scanned using a 3DHISTECH scanning microscope, and images were viewed and selected using Pannoramic Viewer 1.15.3 (3DHISTECH Ltd, Hungary). Vascular thickness was measured and lymphocyte aggregation (LA) were evaluated on H&E staining. For aortic wall, the thickness ≤ 0.2 cm was considered normal (–); the thickness between 0.2 - 0.4 cm was graded as (+); the thickness between 0.4 - 0.6 cm was graded as (++); the thickness over 0.6 cm was graded as (+++). LA was assessed semiquantitatively under 100x magnification: none (-), occasional (+), many (++); and dense clusters (+++). Cells that were double positive for CD68 and HLA-DR were considered as M1 macrophages, while those that were double positive for CD68 and CD163 were regarded as M2 macrophages (15). Positive M1 or M2 cells were counted from ten different squares (about 0.45mm^2^) under 400x magnification, which were randomly selected from the areas with prominent inflammatory infiltrate (16). Different layers of each specimen were counted separately by two raters who were blinded to patients’ clinical data. Average number of the ten squares by the two raters were calculated and used for analysis. Similarly, ten squares (about 0.45mm^2^) within the greatest inflammatory infiltrates were chosen from different layers of each specimen for CCL2 quantification. Image pro-plus 7.0 (Media Cybernetics, Silver Spring, USA) was used in this process and the results were presented as the average ratio of the integrated optical density (IOD) to the area of the selected fields.

### Enzyme-Linked Immunosorbent Assay

Peripheral CCL2 levels were measured in patients at baseline (n=59), at 6 months (n=38) after treatment and in healthy control subjects (n=46). Serum was collected from each patient and control subjects and stored at –80°C. The concentration of CCL2 in blood samples was analyzed using commercial enzyme-linked immunosorbent assay (ELISA) kits (R&D system, DCP00), according to the manufacturer’s instructions.

### Statistical Analysis

Data are expressed as mean ± standard deviation for quantitative data following the normal distribution, as median and interquartile for quantitative data not following the normal distribution, or as frequencies (percentage) for categorical data. Comparisons between patients and controls were performed using the Student’s *t*-test, Mann-Whitney test or chi-square test, as appropriate. Paired Student’s *t*-test was used to compare serum CCL2 levels before and after treatment. Student’s *t*-test was performed to compare peripheral CCL2 levels among patients with different activity status assessed by Kerr criteria (17). Correlation analysis of peripheral CCL2 and other inflammatory indexes including erythrocyte sedimentation rate (ESR), C-reactive protein (CRP), and interleukin 6 (IL-6) were performed using Spearman or Pearson correlation analysis. Receiver operating characteristic (ROC) curve and area under the curve (AUC) were used to evaluate the potential of CCL2 to serve as a biomarker for assessment of patients’ disease activity. ROC was also performed for ESR and CRP and the efficacy between CCL2 and ESR or CRP in this performance was compared. A two-sided p value < 0.05 was considered to indicate statistical significance.

## Results

### Current Treatments Exerting Minimal Effect on Vascular Fibrosis in TA

Vascular specimens from the 15 patients with TA included ascending aorta (n = 12), aortic valve (n = 1), abdominal aorta (n = 1) and renal artery (n = 1). Vascular specimens of the control subjects (n = 4) included normal aorta (n = 1), and renal arteries (n=3). Clinical characteristics of these patients are listed in **Table 1**. There were no significant differences in terms of onset age (35.85 ± 14.14 vs. 39.85 ± 11.81 years, P = 0.95) or disease duration (29.75 ± 23.19 vs. 32.43 ± 40.27 months, P = 0.88) between the treated and untreated patients for vascular tissue study. Based on the Kerr criteria, the majority (6/8, 75%) of untreated patients were in the active status of TA, whereas most treated patients (6/7, 85.71%) were in the inactive status. Levels of CRP and IL-6 were non-statistically higher in untreated patients than in treated patients (CRP: 50.63 ± 67.50 vs. 23.69 ± 40.97 mg/L, P = 0.12; IL-6: 24.99 ± 23.86 vs. 8.14 ± 9.55 pg/mL, P = 0.07).

**Table 1:** Clinical characteristics of the patients enrolled for vascular tissue examination.

Patients	Gender (F/M)	Specimen	Thickness	Onset age (y)	Symptom duration (mo)	Treatment	ESR (mm/h)	CRP (mg/L)	IL-6 (pg/mL)	LA
Untreated patients (n = 8)										
1	F	Ascending aorta	+++	46	16	N	22	3	3.3	+++
2	M	Renal artery	++	42	5	N	80	71.4	13.54	++
3	F	Ascending aorta	+	24	43	N	11	37.8	74.06	++
4	M	Ascending aorta	–	43	62	N	10	1	18.65	+++
5	M	Ascending aorta	+	39	51	N	22	44.8	22.97	++
6	M	Aortic valve	+	56	46	N	7	24.9	34.11	++
7	M	Ascending aorta	+	44	3	N	92	207.4	/	++
8	F	Abdominal aorta	+++	28	12	N	117	14.7	8.33	++
Treated patients (n = 7)										
1	F	Ascending aorta	++	29	24	P + LEF	53	/	5.3	+
2	M	Ascending aorta	+++	33	24	P + CTX	5	1.9	4.8	-/+
3	M	Ascending aorta	++	39	10	P	/	14.7	/	++
4	F	Ascending aorta	+	40	12	P + CTX	79	8.7	27.5	-/+
5	F	Ascending aorta	+++	31	120	P	10	18.4	2.93	+
6	M	Ascending aorta	++	43	1	P + HCQ	36	2.6	5.3	++
7	F	Ascending aorta	++	64	36	P + LEF	35	4	3	+
Control subjects (n = 4)										
Con 1	M	Ascending aorta	–	25	/	/	/	/	/	–
Con 2	M	Renal artery	–	53	/	/	/	/	/	–
Con 3	F	Renal artery	–	39	/	/	/	/	/	–
Con 4	F	Renal artery	–	27	/	/	/	/	/	–

F, Female; M, male; N, no; mo, month; P, prednisone; LEF, leflunomide; CTX, cyclophosphamide; ESR, erythrocyte sedimentation rate; CRP, C-reactive protein; Thickness, ≤ 0.2 cm (-); 0.2 - 0.4 cm (+); 0.4 - 0.6 cm (++); ≥ 0.6 cm (+++). LA (lymphocyte aggregation), none (-), occasional (+), many (++); dense clusters (+++).

**Figure 1:**
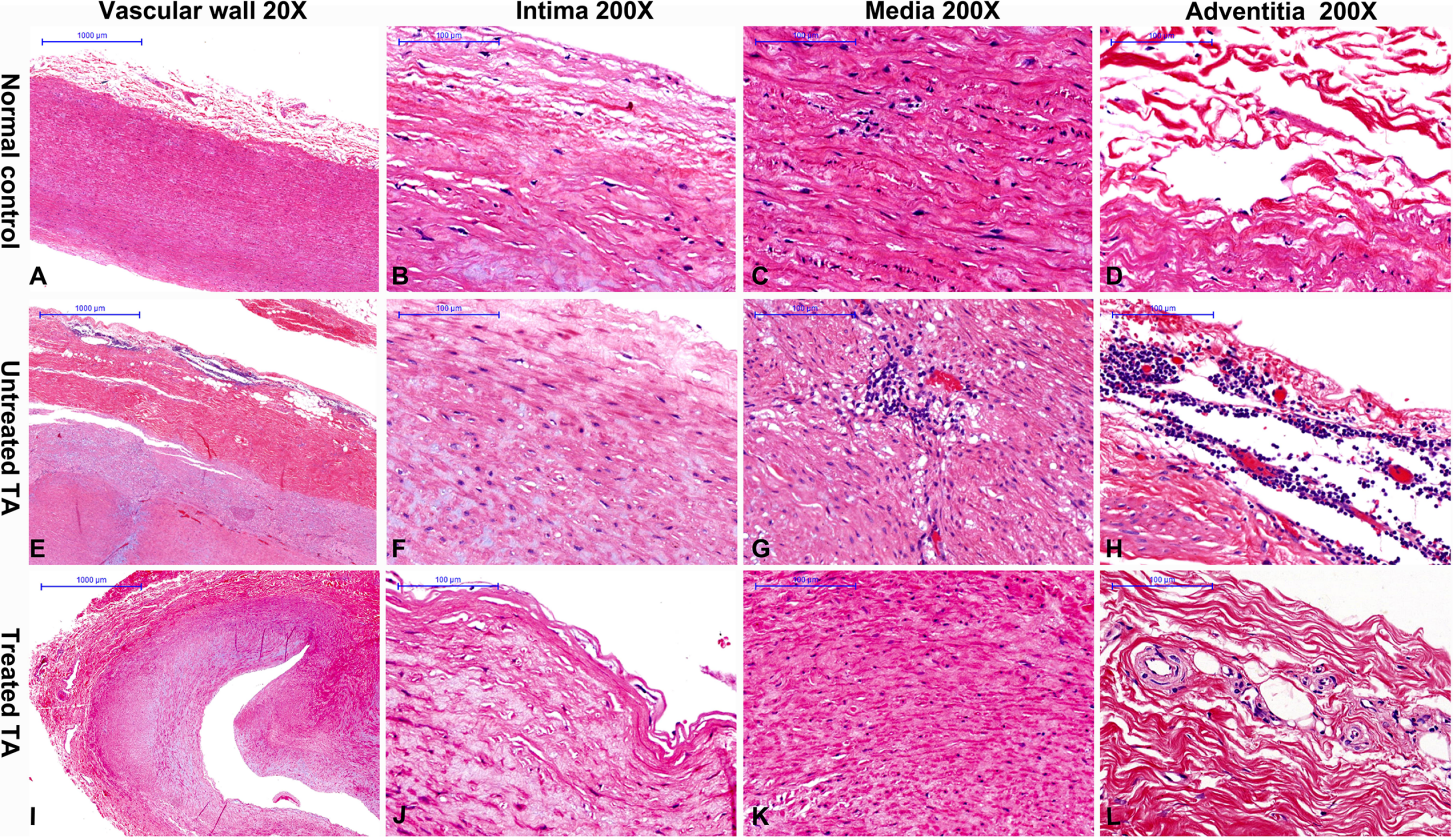
Hematoxylin and eosin staining of vascular tissue from controls, untreated and treated patients with TA **(A)** Representative hematoxylin and eosin images of normal aorta **(A–D)**, untreated TA aorta **(E–H)** and treated TA renal artery **(I–L)**. A great amount of inflammatory cells are observed in untreated patients **(H)**. The inflammatory cells are decreased in treated patients **(I)**.

In contrast to normal vascular tissue (**Figures 1A–D**) H&E staining revealed inflammatory cells to be prominent in all untreated patients (100%, **Table 1** and **Figures 1E–H**), predominantly noted in the vascular adventitia (**Figure 1H**). Vascular wall thickening and fibrosis without excess inflammation were observed in most treated patients (85.71%, **Table 1** and **Figures 1I–L**).

### Increased M1 infiltration in the vascular adventitia of untreated patients

**Figure 2:**
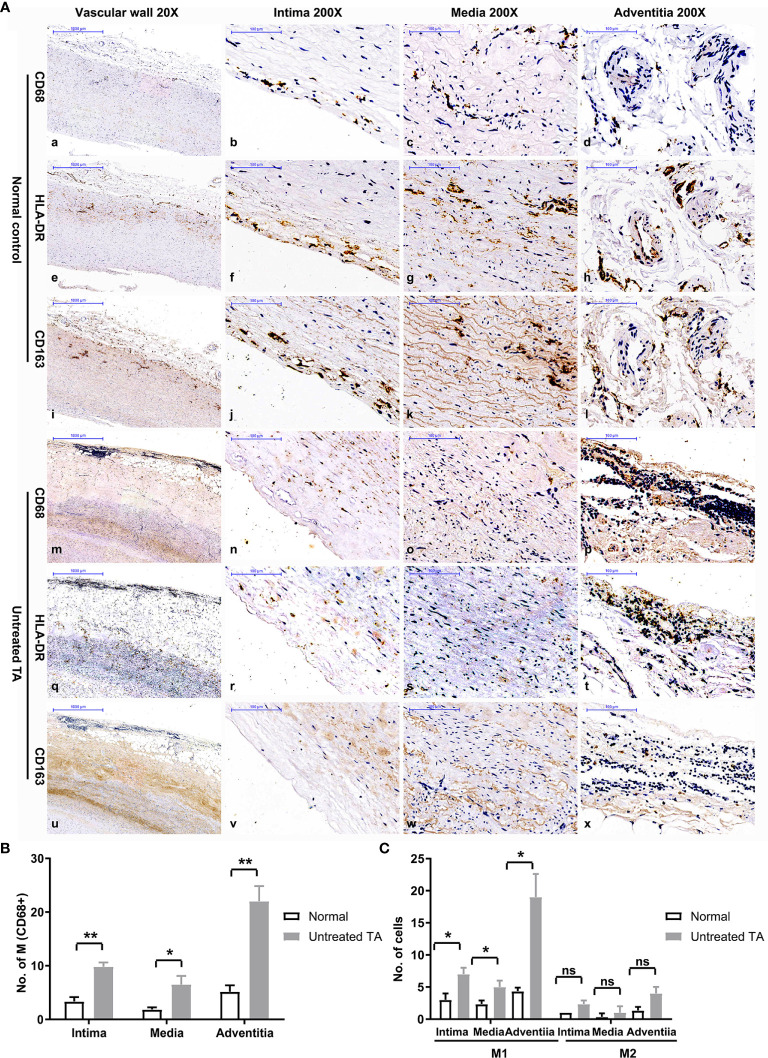
Distribution and phenotype of macrophages in controls and untreated patients with TA. **(A)** Representative immunohistochemistry images of CD68, HLA-DR and CD163 positive cells in control arterial specimens (CD68:a-d; HLA-DR: e-h; CD163: i-l) and TA aortic specimens (CD68: m-p; HLA-DR: q-t; CD163: u-x); image a, e, i, m, q, u-20X, the remaining images-200X. **(B)** The number of macrophages in different layers of control arterial specimens and TA aortic specimens. **(C)** Number of M1 or M2 macrophages in different layers of control specimens and TA aortic specimens. *< 0.05; **< 0.01; ns, no significance.

Significantly higher infiltration of macrophages was observed in all three layers of untreated vascular tissues than in control samples (**Figures 2A, B**). The infiltrating macrophages were identified as M1 owing to their CD68+HLA-DR+ phenotypes (**Figures 2A**q-t, C, P < 0.01). Most macrophages were distributed within the vascular adventitia (**Figures 2A**p, **B**), where the vasa vasorum was enriched and the inflammation was prominent.

### Increased M2 Infiltration in the Vascular Media in Treated Patients

**Figure 3:**
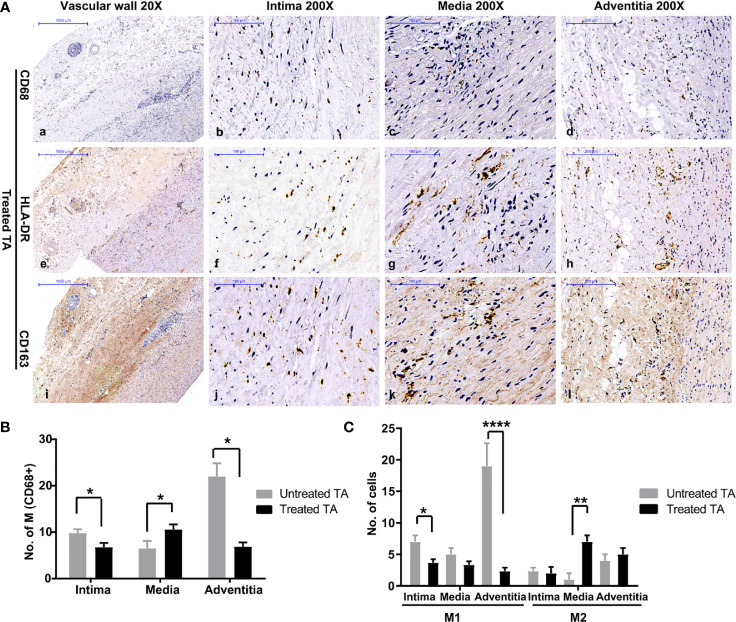
Distribution and phenotype of macrophages in treated patients with TA **(A)** Representative images of CD68 (a–d), HLA-DR (e–h), and CD163 (i–l) positive cells in treated aorta. **(B)** Number of macrophages in different layers of untreated TA arteries, and treated TA arteries. **(C)** Number of M1 and M2 macrophages in vascular intima, media, and adventitia of untreated arteries, and treated arteries. *< 0.05; **< 0.01; ****< 0.0001.

Compared with untreated tissue, macrophage infiltration was significantly lower in treated vascular tissue (**Figures 3A**a–d, **B**). However, analysis of macrophages in different layers of vascular tissue revealed a distinct pattern. Different from adventitia in untreated vascular tissue, macrophages mainly infiltrated in the media of treated vascular tissue (**Figures 3A**k, **B**). Moreover, the macrophages present in treated vascular media were predominantly M2 type (P = 0.002, **Figure 3C**). These results suggest a shift in the phenotype of infiltrated macrophages in vascular lesions from M1 to M2 following treatment for TA.

### Consistent Distribution and Similar Changes of CCL2 as Macrophage in Vascular Lesions

**Figure 4:**
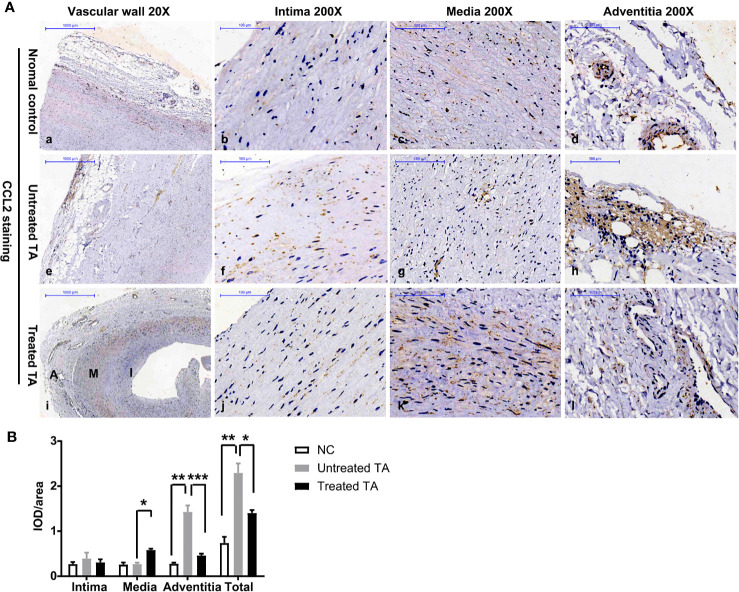
CCL2 expression in arterial specimens from controls, untreated TA, and treated TA **(A)** Representative immunohistochemistry images of CCL2 in normal aorta (a–d), TA untreated aorta (e–h), and TA treated renal artery (i–l). **(B)** Quantification of CCL2 expressions in normal arteries, untreated, and treated TA arteries (intima, media and adventitia). *< 0.05; **< 0.01; ***< 0.001.

Given that CCL2 is a major monocyte chemotactic protein, CCL2 expression in lesion tissue of TA patients was further evaluated. Compared with normal vascular tissue (**Figures 4A**a–d, **B**), CCL2 levels were significantly higher in untreated tissue of patients with TA (**Figures 4A**e–h, P = 0.003). In addition, CCL2 was mainly expressed in the vascular adventitia where macrophage infiltration was high (**Figures 4A**h, **B**, P < 0.01).

In contrast, the total expression of CCL2 throughout vascular tissue was significantly lower in treated patients (**Figures 4Ae, i, B,** P < 0.01), especially in the adventitia where CCL2 expression was decreased (**Figures 4A**h,l, **B**, P < 0.001). However, in the vascular media of treated patients, CCL2 expression was significantly increased and distributed along the tissue cells (**Figures 4A**k, **B,** P < 0.05).

### Correlation of Peripheral CCL2 Levels With Disease Activity in TA

**Figure 5:**
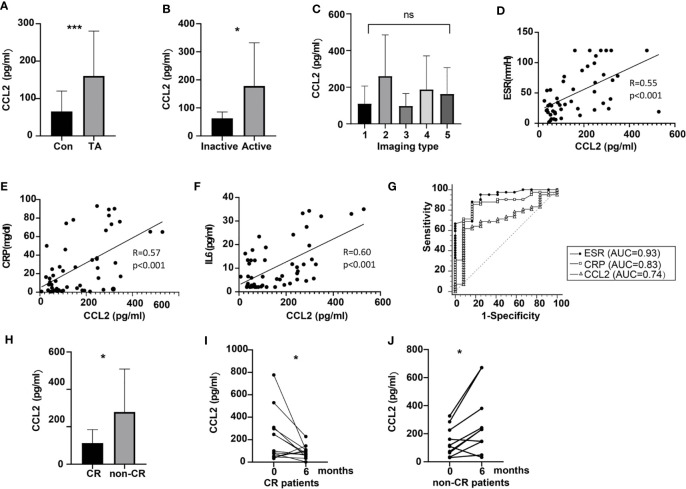
Expression of peripheral CCL2 and the relationship of CCL2 levels with disease activity in patients with TA. **(A)** Levels of peripheral CCL2 in normal controls and patients with TA (n_TA_ = 59, n_control_ = 46, P < 0.001). **(B)** Levels of peripheral CCL2 in active and inactive patients with TA (n_active_ = 46, n_inactive_ = 13, P = 0.01). **(C)** Levels of peripheral CCL2 in patients with different imaging types (n_type I_ = 20, n _type II_ =8, n_type III_ = 3, n _type IV_ =2, n _type v_=26). **(D–F)** Relationship between CCL2 levels and ESR, CRP or IL-6 levels in patients with TA (n = 59) **(G)**. ROC of CCL2, ESR and CRP to predict disease activity in patients with TA (n = 59). **(H)** CCL2 levels in CR and non-CR patients (n_CR_ = 24, n_non-CR_ = 14, P = 0.02). **(I)** Changes of peripheral CCL2 levels in CR patients at baseline and the 6^th^ month after treatment (n = 24, P = 0.04). **(J)** Changes of peripheral CCL2 levels in non-CR patients at baseline and the 6^th^ month after treatment (n = 14, P = 0.02) AUC, area under the curve; CR, clinical remission; ESR, erythrocyte sedimentation rate; CRP, C-reactive protein, IL-6, interleukin 6; CCL2, chemokine (C-C motif) ligand 2; ROC, receiver operatic characteristic; TA, Takayasu arteritis; *< 0.05; ***< 0.001; ns, no significance.

Clinical characteristics of patients for whom serum estimation of CCL2 was performed are shown in **Table 2**. The results show that serum CCL2 level was remarkably and significantly higher in patients with TA than in healthy controls (160.30 ± 120.05 vs. 65.58 ± 54.56 pg/mL, P < 0.001, **Figure 5A**). Among patients with TA, serum CCL2 level was significantly higher in active patients (Kerr ≥ 2) than in inactive patients (178.20 ± 153.96 vs. 62.90 ± 22.74 pg/mL, P = 0.01, **Figure 5B**). No significant difference was observed in the serum CCL2 levels among patients with different imaging types (P > 0.05, **Figure 5C**).

**Table 2:** Baseline clinical characteristics of the patients recruited for serum detection.

Characteristics	TA group (N = 59)	Healthy group (N = 38)	P-value
**Age (y, mean ± SD)**	35.62 ± 14.03	38.04 ± 10.94	0.34
**Gender ratio (F:M)**	49:10	22:7	0.79
**Disease duration (mo)**	41.51 ± 96.29	/	**/**
**Active status (n, %)**	46 (77.97)		
**Headache/Dizziness (n, %)**	30 (50.8)	/	
**Fever (n, %)**	13 (22.00)	/	
**Weakness (n, %)**	18 (30.5)	/	
**Hypertension (n, %)**	20 (33.9)	/	
**Pulselessness/decreased pulse (n, %)**	16 (27.1)	/	
**Neck murmur (n, %)**	16 (27.1)	/	
**Hemoglobin (g/L)**	114.50 ± 16.97	110−150 (F), 120−160 (M)	**/**
**WBC (× 10^9^/L)**	8.95 ± 7.50	3.5−9.5	**/**
**PLT (× 10^9^/L)**	281.14 ± 88.42	100−300	**/**
**ESR (mm/H)**	47.86 ± 39.13	< 28 (F), < 40 (M)	**/**
**CRP (mg/L)**	29.71 ± 40.01	< 3	**/**

WBC, white blood cell; PLT, platelet; ESR, erythrocyte sedimentation rate; CRP, C-reactive protein.

Erythrocyte sedimentation rate (ESR), CRP, and IL-6 are common indicators of disease activity in TA. Correlation between peripheral CCL2 and these parameters were further evaluated. The results suggested that serum CCL2 levels were moderately correlated with ESR, CRP, and IL-6 (P < 0.001 for all; **Figures 5D–F**, respectively). To evaluate the efficacy of CCL2 to predict disease activity (Kerr ≥ 2), ROC curve analysis was performed. The results indicated that CCL2 cut-off value of 100.36 pg/mL was able to predict disease activity in TA (AUC as 0.74) with specificity of 91.67% and sensitivity as 51.78% (P = 0.03, **Figure 5G**). But its efficacy was lower than ESR (ESR cut-off value = 20mm/H, AUC = 0.93, Sensitivity = 88.37%, Specificity = 84.62%, p < 0.01; CCL2 vs. ESR: P = 0.03, **Figure 5G**). No significant difference was observed between CCL2 and CRP in this performance (CRP cut-off value = 2.1mg/dL, AUC = 0.83, Sensitivity = 86.05%, Specificity = 76.92%, P < 0.001; CCL2 vs. CRP: P = 0.23, **Figure 5G**).

After the treatment, patients’ disease status was evaluated. At 6 months, 15 patients were excluded due to lacking serum samples, whereas the other 6 patients were lost to follow-up. Thus, 38 patients were remained at 6 months. The average prednisone dose for them was 14.63 ± 4.84 mg per day. Among 38 patients, 24 (63.16%) patients achieved CR. Among the CR patients, the percentage of those with CCL2 of less than 100.36 pg/mL was 83.33%, while among non-CR patients, the percentage of those with CCL2 more than 100.36 pg/mL was 85.71%. Compared to the patients with CR, CCL2 levels in non-CR patients were higher at 6 months (88.71 ± 55.89 vs. 279.49 ± 229.46pg/mL, P = 0.02, **Figure 5H**). Moreover, compared to their baseline levels, CCL2 serum levels in CR patients were decreased (220.18 ± 222.69 vs.88.71 ± 55.89 pg/mL, P = 0.04, **Figure 5I**), whereas those in non-CR patients were increased after 6 months (142.45 ± 104.76 vs. 279.49 ± 229.46 pg/mL, P = 0.02, **Figure 5J**).

## Discussion

The present study investigated the potential role of macrophage and CCL2 in the vascular pathogenesis of TA. The distribution pattern and phenotype of macrophages and CCL2 expression in vascular tissue of patients with TA indicated that macrophage presented a phenotype shift (M1 to M2) and distribution change (adventitia to media) as the disease progressed from an active to an inactive phase. Vascular CCL2 expression was closely related with macrophage distribution during this process. Moreover, peripheral blood CCL2 levels were found to be elevated and correlated with patient disease activity, thus serving as a promising biomarker in the evaluation of patient treatment efficacy.

Macrophages are known to contribute to the progression of vascular lesions in TA (16). It is well known that macrophages with different phenotype can present distinct biological activity (18). M1 subset is believed to play a pro-inflammatory role by secreting IL-1, IL-6 and tumor necrosis factor-α (TNF-α), whereas M2 subset promote tissue fibrosis by producing pro-fibrotic factors such as transformation growth factor-β (TGF-β) (8, 19–22). Based on this concept, an M1-dominated macrophage population is expected to enhance vascular inflammation in the acute stage, while an M2-dominated macrophage population should contribute to vascular fibrosis in chronic stage in TA.

Excessive polarization of macrophages to M1 or M2 in different stages of disease implies an imbalance of macrophage differentiation regulators in vascular lesions of TA. As previously reported (14), multiple pro-inflammatory cytokines were detected in active lesions of TA, such as interferon-γ (IFN-γ), IL-6, IL-12, and IL-17. CD4^+^ T cells also mainly presented as pro-inflammatory phenotypes, Th1 and Th17. These factors probably promoted differentiation of macrophages to M1 subset in acute stage. However, mechanism of M2 polarization in the chronic stage is poorly understood. Common M2 polarization cytokines such as IL-4, IL-10 and IL-13 were less frequently observed in vascular lesions (14). In the present study, the chronic stage vascular specimens were obtained from patients post treatment (mainly glucocorticoids and different immunosuppressants). Thus, the impact of medications on macrophage M2 polarization cannot be ruled out; this, however, further research is needed to validate this possibility.

Based on these observations, application of precise treatment strategies targeting M1 and M2 in acute and chronic stages, respectively, is expected to improve patient vascular lesions more effectively. Although current therapies have impacts on macrophage phenotype, they are not able to prevent vascular remodeling process. Thus, there is a need for exploring novel treatment regimens that can inhibit inflammation as well as fibrosis.

In the present study, we observed that CCL2 was expressed at the same sites as macrophage infiltration in the vascular lesions (despite the stage of disease), indicating the critical role of CCL2 in macrophage recruitment. CCL2 could be produced by immune cells as well as tissue cells such as myofibroblasts and smooth muscle cells (5, 23, 24). In a previous study, we have also shown that CCL2 can also be expressed by adventitial fibroblast after IL-6 stimulation (25). Therefore, multiple cells may be involved in macrophage recruitment *via* CCL2 expression in vascular lesions of TA.

In addition to the chemotactic function of CCL2, research has shown that CCL2was also involved in tissue fibrosis. It has been reported that CCL2 was able to promote proliferation and IL-6 production of vascular smooth muscle cells (26). In addition, CCL2 is reportedly capable of inducing collagen synthesis and TGF-β expression in lung fibroblasts (27). Since vascular lesions in TA are characterized by fibrosis in chronic stage, these studies suggest that CCL2 may play multiple roles in TA pathogenesis.

In the present study, we observed that peripheral CCL2 levels were correlated with Kerr score as well as with disease activity parameters such as ESR and CRP in TA. In vascular tissues, CCL2 was closely related to macrophage infiltration, thus peripheral CCL2 level may also reflect vascular tissue CCL2 expression and macrophage infiltration indirectly. Its levels were found to be significantly different in the same patient during disease progression, as well as in patients showing remission vs. those that did not show remission. Therefore, CCL2 may serve as a promising biomarker in the assessment of disease activity and treatment effect.

This was a preliminary study of macrophages and CCL2 in TA. More detailed studies such assessing the specific phenotypes of macrophage in vascular tissue such as M2a, M2b, and M2c, are required. In addition, due to the low incidence of this disorder, the tissue sample size in this study was relatively small. Further research, with larger cohort of patients, is warranted to validate the results of this study.

## Conclusion

Macrophages contribute to vascular pathological changes in TA by undergoing phenotype transformation and distribution changes. CCL2 is an important factor for recruiting macrophages and a potential biomarker for disease activity.

## Data Availability Statement

The original contributions presented in the study are included in the article/**Supplementary Material**. Further inquiries can be directed to the corresponding author.

## Ethics Statement

The studies involving human participants were reviewed and approved by Institutional Review Board of Zhongshan Hospital, Fudan University, China. The patients/participants provided their written informed consent to participate in this study.

## Author Contributions

XK was responsible for the data analysis and manuscript writing. MX provided the control vascular samples. XC participated in the IHC staining. LYM was responsible for collecting the clinical data of patients for serum CCL2 detection. HC and JH were responsible for the evaluation of macrophages from vascular specimens. XS provided part of control vascular specimens. LLM helped the statistical analysis. LJ designed the study. All authors contributed to the article and approved the submitted version.

## Funding

This work was supported by the National Natural Science Foundation of China (grant number 81771730, 81801598), Science and Technology Commission of Shanghai Municipality (20YF1406800, 17140902000), China Postdoctoral Science Foundation (2020M671008), and the Youth Research Fund of Zhongshan Hospital, Fudan University (2020ZYYS-001).

## Conflict of Interest

The authors declare that the research was conducted in the absence of any commercial or financial relationships that could be construed as a potential conflict of interest.
