# Supplementary material for: Potential Role of Macrophage Phenotypes and CCL2 in the Pathogenesis of Takayasu Arteritis
Source: Front Immunol. 2021 May 17;12:646516. doi: 10.3389/fimmu.2021.646516 (PMC8165246; doi:10.3389/fimmu.2021.646516)
Supplement: Supplementary file 1 [file DataSheet_1.doc]

**
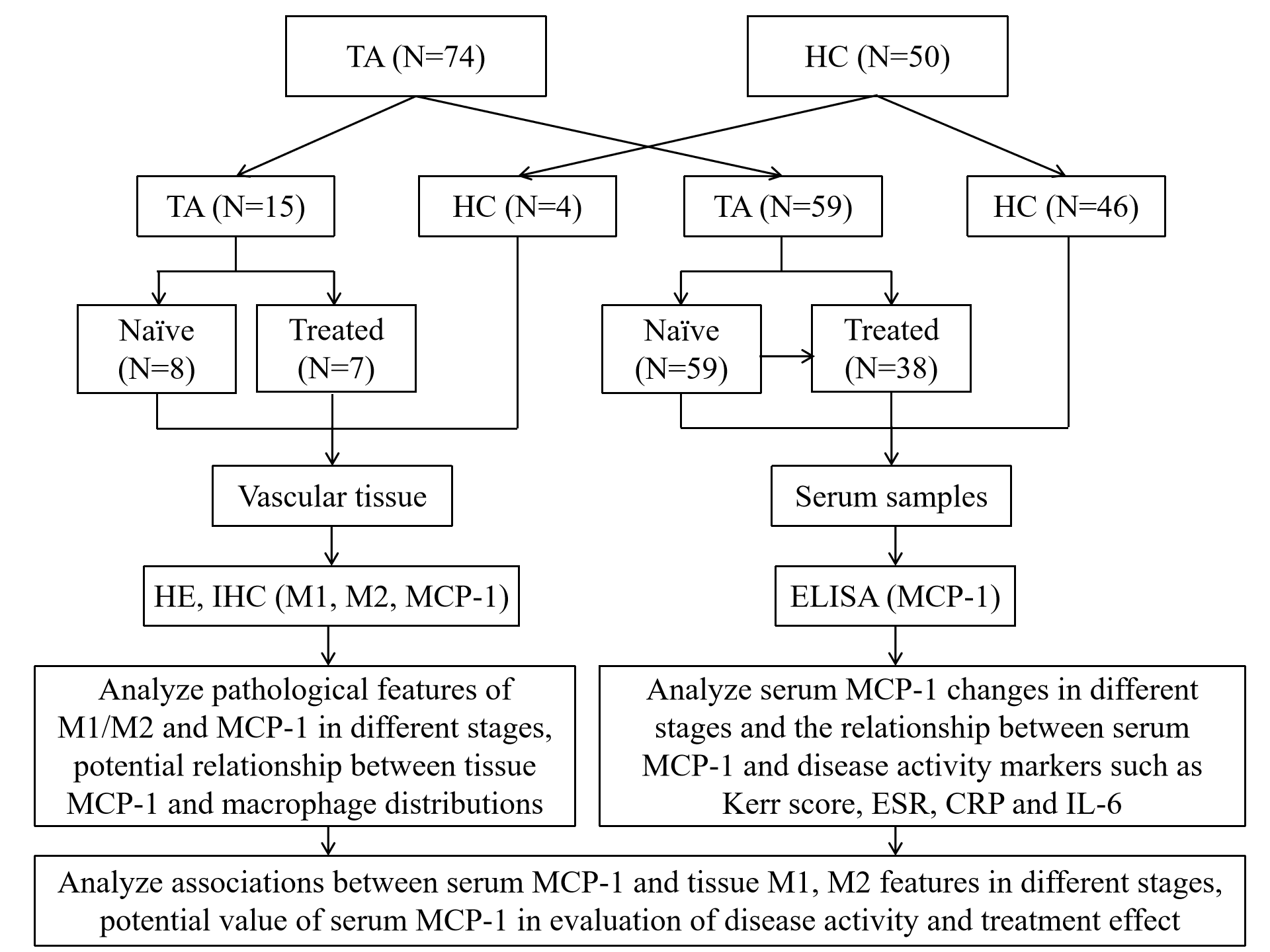
**

**Supplementary Figure 1. Flow chart of this study**

Patients were recruited to detect macrophages phenotype, distribution, and MCP-1 expression in vascular tissue and MCP-1 levels in peripheral blood of naïve and treated TA patients.

TA: Takayasu arteritis; HC: healthy controls; IHC: immunohistochemistry; ELISA: enzyme- linked immunosorbent assay; MCP-1: monocyte chemoattractant protein-1
